# S8. RELATIONSHIP BETWEEN ALLOSTATIC LOAD AND POOR FUNCTIONAL CAPACITY IN YOUTH AT ULTRA-HIGH RISK FOR PSYCHOSIS

**DOI:** 10.1093/schbul/sby018.795

**Published:** 2018-04-01

**Authors:** Maximus Berger, Suzie Lavoie, Patrick D McGorry, Barnaby Nelson, Connie Markulev, Hok Pan Yuen, Miriam Schaefer, Zoltan Sarnyai, G Paul Amminger

**Affiliations:** 1 James Cook University; 2 Orygen, The National Centre of Excellence in Youth Mental Health, University of Melbourne; 3 Orygen, The National Centre of Excellence in Youth Mental Health

## Abstract

**Background:**

Current pathophysiological models of psychotic disorders suggest that stress contributes to the aetiology and trajectory of the disorder. Allostatic load (AL), a multisystem index of immune, neuroendocrine and metabolic dysregulation, is thought to represent the cumulative biological impact of stress. Two recent studies suggest that AL is elevated in patients with first-episode psychosis and related to psychotic symptoms and poor social and occupational functioning. Here, we investigate the relationship between AL and clinical outcomes in individuals at ultra-high risk for psychosis.

**Methods:**

AL was measured in a sub-group of participants of the NEURAPRO study, a multicentre randomized-controlled trial of omega-3 polyunsaturated fatty acids versus placebo in people aged 13 - 40 at UHR for psychosis. A total of 106 participants who underwent additional biomarker analysis were included in the present study. Biomarkers for the AL index were selected based on (1) representation of several physiological systems including the cardiovascular, neuroendocrine, immune, and metabolic systems, (2) use in previous AL research, and (3) associations with disease risk. We adopted a scaled AL algorithm whereby each marker proportionally contributes to the overall AL index. Clinical outcomes were assessed 3 and 12 months after study intake using the Social and Occupational Functioning Assessment Scale (SOFAS), the Brief Psychiatric Rating Scale (BPRS), the Scale for the Assessment of Negative Symptoms (SANS), the Montgomery-Asberg Depression Rating Scale (MADRS) and the Young Mania Rating Scale (YMRS). We hypothesised that AL would be (1) associated with higher symptoms scores and reduced functioning at baseline and (2) related to more severe symptoms and reduced functioning at the 3 and 12 month assessments. These hypotheses were tested by calculating Pearson correlation coefficients and by using linear regression modelling, respectively.

**Results:**

No significant correlations of AL with any of the psychometric scales are observed at study intake. AL at baseline was associated with lower SOFAS scores at 3 months (B=-1.436, p=0.042) but not at 12 months (B=-1.096, p=0.297). No prospective associations of AL were found with any of the other psychometric measures (all p>0.05).

**Discussion:**

Our data support the notion that multisystem dysregulation, indexed as AL, may be a potential predictor of early treatment response and warrants further investigation. These observations are consistent with recent research demonstrating elevated AL in patients with psychotic disorders that is related to reduced functioning.

